# Potassium Intake and Human Health

**DOI:** 10.3390/nu16060833

**Published:** 2024-03-14

**Authors:** Lanfranco D’Elia

**Affiliations:** Department of Clinical Medicine and Surgery, “Federico II” University of Naples Medical School, 80131 Naples, Italy; lanfranco.delia@unina.it

Potassium is a monovalent cation widely present in nature, where it is not in metallic form, but always in combination with other substances, especially chloride. The main sources of dietary potassium are fruit and vegetables, where it is usually combined with organic acids (e.g., potassium citrate). In the body, a large part of the potassium pool is localized in the muscles and the skeleton, but also in the central nervous system, intestines, liver, lungs, and skin. Extracellular potassium plays a key role in the regulation of cellular membrane potential and thus in the regulation of nerve and muscle activities [1]. Since potassium is a major intracellular cation, the mechanisms that modulate the gradient between intra- and extracellular content are finely adjusted at different levels [1]. Given this assumption, the effect of potassium on the human body is broad, involving all cells and tissues, although most of the evidence points particularly to its role in the cardiovascular system. Several studies have found an inverse relationship between potassium intake and blood pressure [2,3], with a number of investigations showing the favourable effect of a potassium-rich diet on cardiovascular disease, in part independently of its effect on blood pressure [4,5,6].

In this context, international guidelines suggest a high dietary potassium intake as one of the most recommended lifestyle modifications for the prevention and management of cardiovascular risk [7,8], although there is no general agreement on the threshold of dietary potassium intake at the population level [9,10,11].

Against this background, this Special Issue focusses on new evidence and systematic reviews of the current literature regarding the relationship between potassium intake and human health, in particular in relation to cardiovascular diseases, aiming to (1) add to the knowledge on this topic, (2) support the campaigns to increase dietary potassium consumption for the primary and secondary prevention of cardiovascular diseases, and (3) determine the appropriate potassium intake thresholds to prevent non-communicable diseases. The papers included in this Special Issue mainly explored the effect of potassium intake on cardiovascular risk factors (i.e., diabetes and endothelial function), the interaction between renin–angiotensin–aldosterone system (RAAS) inhibitors and urinary potassium excretion in chronic kidney disease (CKD) patients, and the applications of artificial intelligence (AI) in this field.

The first of the two included meta-analyses assessed the association between habitual potassium intake and the risk of type 2 diabetes [12]. The pooled analysis included seven prospective studies (31,873 participants overall and 4320 new diabetes cases), with the length of follow-up ranging from 4.7 to 20 years. The main results of this meta-analysis show that the habitual dietary potassium intake is associated with the risk of type 2 diabetes in the general population, exhibiting a J-shape relationship. Particularly, apparent benefits regarding slowing the development of type 2 diabetes were noted at consumption levels between 3000 and 5000 mg per day (assuming that 1 mmol = 39 mg and that approximately 70% of the potassium ingested is excreted in urine). The results are supported by the Grade categorization, which detected a moderate quality, and are further strengthened by the stringent inclusion criteria and the relatively large number of participants. Additionally, in support of this relationship, some investigations found a favourable effect of potassium intake on insulin sensitivity [13,14].

Several experimental studies suggest that a high-potassium diet may exert a favourable effect on cardiovascular risk by increasing the production of endothelial nitric oxide [15] and suppressing reactive oxygen species [16]. In this context, another meta-analysis included in this Special Issue evaluated the effect of potassium supplementation on endothelial function (an early predictor of cardiovascular diseases [17]) [18]. Five intervention studies (eight cohorts overall; 332 participants; length of intervention 6 days–6 weeks) were included in this pooled analysis. Potassium supplementations and controls consisted of capsules or diets, and 24 h urine collection was used as a proxy for potassium intake. Endothelial function was assessed via flow-mediated vasodilation (FMD)—a widely used tool for clinical studies [17]—where reduced FMD was associated with conditions predisposing to atherosclerosis and cardiovascular disease. The main results of this meta-analysis suggest that potassium supplementation is associated with an improved endothelial function. A positive association was detected between the amount of potassium supplemented and its effect on FMD; i.e., a higher potassium intake was associated with greater vasodilation. In particular, this effect was greater at potassium excretions above 90 mmol/day. The major strengths of this meta-analysis are the high overall quality of the evidence utilising the Grade assessment approach, and the inclusion of only intervention trials with an exclusive evaluation of potassium intake.

Notably, guidelines on CKD management [19] highlight the role of dietary potassium intake in CKD, recommending the assessment of dietary potassium intake in CKD patients, particularly those with potential hyperkalaemia [20]. In this context, two other studies included in this Special Issue explored different aspects of the assessment of dietary potassium intake in patients with CKD [21,22]. Moreover, since the mechanisms of potassium homeostasis and excretion are impaired in these individuals, 24 h urine collection (the recognised gold standard for monitoring potassium intake [23]) may not be reliable to evaluate potassium intake in this setting.

In particular, one study evaluated the relationship between urinary potassium excretion (via 24 h urine collection) and dietary potassium intake (via a questionnaire) in a cohort of stage 3–4 CKD patients with or without RAAS inhibitor therapy [21]. The main analysis, which included 138 patients (60 ± 13 years), indicated that urinary potassium excretion was significantly, but weakly, associated with the glomerular filtration rate and dietary potassium intake. Moreover, serum potassium levels were not associated with dietary potassium intake, while an inverse relationship was detected with the glomerular filtration rate. On the other hand, after stratifying by RAAS inhibitor therapy, a direct association between urinary potassium excretion and dietary potassium intake was confirmed only in patients who were not on RAAS inhibitor treatment, while the relationship between serum potassium and renal function was confirmed in both groups. Thus, although urine potassium excretion can be considered an indicator of potassium intake in individuals with or without an altered kidney function, RAAS inhibitor therapy seems to affect the relationship between urinary potassium excretion and dietary potassium intake, at least in patients with stages 3–4 CKD.

Another study included in this Special Issue also explored the assessment of dietary potassium intake in CKD patients using Bayesian networks derived from AI [22]. A total of 375 adults with CKD were included in an analysis aimed to develop a clinical tool to estimate potassium intake using 24 h urinary potassium excretion. From a total of 25 physical and dietary characteristics selected to evaluate potential associations with urinary potassium excretion, 14 were employed to improve the ergonomics of the model and make it usable in clinical settings. The main analysis showed that the parameters most correlated with 24 h urinary potassium were weight, height, age, meal portion, and glomerular filtration rate. In particular, the results highlighted that the expression of potassium intake was more associated with clinical characteristics and renal function than the potassium content of the ingested food. This finding may explain the poor agreement between the data on potassium intake in dietary surveys and 24 h urine collection, which is affected by potassium-rich foods. Therefore, the tool may be more suitable for estimating potassium consumption than a simple questionnaire on patients’ dietary habits and may be an ergonomic and user-friendly application for CKD patients to evaluate their potassium consumption and increase compliance with dietary recommendations. Notably, the results of this study were limited by a single 24 h urine collection and the lack of external validation, although the internal validation had a 74% accuracy level.

Lastly, as for AI, one of the papers in this Special Issue evaluated the cardiovascular and renal outcomes in patients with hyperkalaemia using machine learning models [23]. Potassium intake has a non-univocal role in CKD patients: indeed, while dietary potassium restrictions may lead to benefits for high-risk hyperkalaemic patients, they may lead to an unhealthy diet and the beneficial effects of dietary potassium intake may be lost [12,18,24] due to a reduction in plant-based food intake. In addition, this type of diet (i.e., low consumption of fruit and vegetables and high protein intake) would not counteract the increased net endogenous acid production (NEAP) due to diet [19], which in turn is associated with the progression of kidney damage [25]. Kanda et al. reported the development and validation of risk prediction models using machine learning technologies to detect hyperkalaemic patients at high risk of mortality, as well as cardiovascular and renal outcomes, over a three-year period after their first hyperkalaemic episode [23]. A total of 24,949 patients with hyperkalaemia were included in the model derivation and internal validation, and 86,279 patients with similar characteristics were included for external validation. The main findings of this study suggest a similar predictive role of clinical variables in different outcomes. In particular, age, CKD stage, glomerular filtration rate, and history of emergency room visits were among the most relevant variables. In addition, the use of some drugs (e.g., loop diuretics, heparin, and sodium bicarbonate), RAAS inhibitor discontinuation within one year from the hyperkalaemic episode, and some laboratory data, including triglyceride, glycosylated haemoglobin, and brain natriuretic peptide levels, were within the top 20 most important variables across all outcomes. Hence, these results indicate a possible use of machine learning models for real-world risk assessments in patients with hyperkalaemia. Although the model was tested on an external database, further studies are warranted to improve the model’s applicability in different settings.

In conclusion, this Special Issue highlights the complex role of potassium intake in health in different settings and scenarios. In particular, the articles underscore the benefit of a higher potassium intake on two major cardiovascular risk factors (i.e., diabetes and endothelial dysfunction), the potential bias of potassium intake assessments in CKD patients undergoing RAAS inhibitor therapy, and the potential applications of AI in this context. It is suggested that particular attention be paid to potassium assessments in patients with CKD, which would offer insights into potentially simple means to avoid hyperkalaemia and to avoid the need to limit the intake of plant-based foods, which are crucial for cardiovascular prevention. Therefore, the main findings of the available literature support the international recommendations on the increase in daily potassium intakes through regular fresh fruit and vegetable consumption in the general population [9,10,11], and are thus clearly in agreement with the benefits of the model of planetary diet conceived by the EAT-Lancet Commission [26].

However, despite the compelling evidence of the benefits associated with a higher potassium intake and the educational campaigns in favour of a potassium-rich diet to attain the World Health Organization recommendations of a target daily dietary intake of at least 90 mmol of potassium [9], in most countries worldwide, the habitual average of potassium intake is largely below this level [27]. A few approaches have been adopted to achieve this daily intake with reasonable results, for instance, the use of potassium-enriched salt substitutes (75% sodium chloride and 25% potassium chloride) in place of sodium chloride salt (100% sodium chloride), also associated with a reduction in cardiovascular events in large populations [28], or the use of potassium supplementation with slow-release potassium salts, which are generally safe at a low dose [29]. Of note, hyperkalaemia following excessive dietary potassium intake is rare in healthy individuals, and it is more likely to occur in individuals with compromised renal functions [19] or very high intakes of oral potassium supplements.

Nevertheless, further data are needed to reach firm conclusions on the relationship between potassium supplementation and the cardiovascular event rate. Proper intervention studies on the effects of long-term dietary potassium consumption or potassium supplementation are warranted to confirm these trends and allow for the detection of appropriate potassium intake thresholds to prevent cardiovascular diseases in different settings (e.g., the general population, patients with CKD or undergoing pharmacological treatment that affects potassium handling) and allow to improve our knowledge of its effect on non-cardiovascular diseases, such as nephrolithiasis, bone disease, and altered glucose metabolism.
